# Two new species of *Hoya* R.Br. (Apocynaceae, Asclepiadoideae) from Borneo

**DOI:** 10.3897/phytokeys.53.5079

**Published:** 2015-07-21

**Authors:** Michele Rodda

**Affiliations:** 1 The Herbarium, Singapore Botanic Gardens, 1 Cluny Road, 259569 Singapore

**Keywords:** Bako National Park, kerangas, limestone flora, Malaysia, Sabah, Sarawak, taxonomy

## Abstract

Two new *Hoya* R.Br. species from Borneo are described and illustrated. The first, *Hoya ruthiae* Rodda was collected in Sabah on Bukit Baturong, a limestone outcrop. It is one of the few species in the genus to have clear exudate. It is compared with the morphologically related *Hoya uncinata* Teijsm. and Binn. The other, *Hoya bakoensis* Rodda, was collected in the kerangas forests of Bako National Park. It belongs to Hoya section Acanthostemma (Bl.) Kloppenb., a section with numerous members in the Philippines but under-represented in Borneo.

## Introduction

*Hoya* R.Br. is a large and complex genus with more than 500 published species names (IPNI 2015). Kleijn and van Donkelaar (2001) estimated that these names might represent 200–300 species. However their estimate was very conservative. Since 2001 more that 200 new *Hoya* names have been published (IPNI 2015) and the species number estimate for the genus may therefore now lie in between 350 and 450, taking into account a large number of synonyms expected in a horticulturally significant genus. The genus is particularly diverse in the island of Borneo where Nutt (2001) listed 21 species while more recent research bring up the number to an estimated 60–70 species for Sabah alone (Lamb et al. 2014). In Brunei a preliminary checklist comprises 27 species including three undescribed taxa (Rodda 2014).

Numerous papers have been recently published documenting new *Hoya* species from Borneo (Green and Kloppenburg 2014; Rodda and Nyhuus 2009; Rodda and Simonsson 2010; 2011a; b; Rodda and Simonsson Juhonewe 2013a; b; Rodda et al. 2011; 2014a; b; Trân et al. 2011). More notably, Lamb et al. (2014) published eight new taxon names in the genus *Hoya* all described based on materials from Sabah originally collected sterile and brought into cultivation at Kipandi Park (Kampung Kipandi, Moyog, Sabah) where they bloomed and were identified as new taxa. An extensive introduction on the morphology and ecology of Bornean *Hoya* is also found in Lamb et al. (2014).

Two further new *Hoya* species from Borneo are here described. The first, collected by Ruth Kiew in Sabah, is also widely available in cultivation, and is here named *Hoya ruthiae* Rodda. The second was collected by the author in March 2015 during an expedition to Bako National Park (Sarawak, Malaysia) to which it is named after.

## Species treatments

### Hoya ruthiae

Taxon classificationPlantaeGentianalesApocynaceae

Rodda sp. nov.

urn:lsid:ipni.org:names:77148380-1

Figs 1 , 2

#### Diagnostic characters.

Similar to *Hoya uncinata* Teijsm. and Binn as both species have clear exudate, deeply lobed rotate corolla and corpusculum of the pollinarium almost as large as the pollinium. The flattened corolla is smaller (1.5–1.7 cm in diameter) with ovate lobes in *Hoya uncinata*, while the corolla of *Hoya ruthiae* is 1.8–2.2 cm in diameter with narrowly lanceolate lobes.

#### Type.

Malaysia, Sabah, Lahad Datu, Bukit Baturong, on limestone, 7 July 2000, *Kiew R RK5029* (SING, holotype; barcode SING0077484).

#### Description.

Delicate lithophytic climber with clear exudate in all vegetative parts; all vegetative parts glabrous. Leafy *stems* cylindrical, slender, up to 4 mm in diameter, dark brown or grey, with membranaceous peeling bark; internodes 5–15 cm long. *Petioles* terete, fleshy, 5–15 × 1.5–3 mm in diameter, *lamina* lanceolate, fleshy, (5–)7–15 × 1.5–3 cm, apex acute-acuminate, base cuneate, light green above turning red in bright light with numerous grey spots, lighter green underneath; penninerved, secondary veins obscure. *Inflorescences* pseudo-umbelliform or globular, 3–4 cm in diameter, 4–20 flowered; *peduncles* persistent, extra-axillary, terete, 1–3 cm × 1.5–2.5 mm in diameter, glabrous; *pedicels* 10–15 mm × 0.5–0.8 mm in diameter, glabrous. *Buds* conical with a 5-ridged base, ca. 10 × 6 mm. *Calyx* lobes triangular, white-pink 1.3–1.5 × 0.7–1 mm, apex rounded, glabrous; basal *colleters* 1 in each sepal sinus, ovoid. *Corolla* rotate, deeply lobed, 1–1.5 cm in diameter, 1.8–2.2 cm when flattened, white tinged pink, thinly and minutely pubescent inside, outside glabrous, tube 1.5–2 mm long; *corolla lobes* narrowly lanceolate with a triangular acuminate apex, 9–10 × 3–4 mm, laterally revolute, lobe tips recurved. *Gynostegium* stalked, corona column conical 1–1.2 × ca. 2 mm diam, glabrous; *corona* staminal, 2.5–3 mm high, 6–7 mm in diameter, fleshy, yellow with a purple centre; *corona lobes* laterally compressed, ovate above, with revolute margins beneath, 2.8–3.2 mm × 1–1.2 mm, inner process erecto-patent, linear with an acute tip, as high as the anthers, outer process round. *Anthers* ovate, 650–750 × 300–400 µm, with apical round membranaceous appendage as high as the style-head apex. *Pollinia* oblong, with obliquely truncate apex and round base and evident pellucid margin, 550–630 × 150–200 μm; *caudicles* attached at the base of the retinaculum, elongate, ca. 150 µm long, *corpusculum* 600–650 × 250–300 μm; style-head 5-angled in cross section, with 5 spreading lobes alternating with the stamens; *style-head* apex columnar, 1–1.2 mm long, ca. 0.5 mm broad at the base, apex conical; *ovary* linear, ca. 2 mm long, each carpel ca. 0.5 mm wide at the base. *Fruits* and *seeds* unknown.

#### Etymology.

This species is named after Ruth Kiew (1946–), tropical botanist based at the Forest Research Institute Malaysia and author of numerous publications on *Begonia* L. and Gesneriaceae, among others.

#### Distribution and ecology.

*Hoya ruthiae* is only known from Bukit Baturong, Sabah, Malaysia, where it was found growing on limestone.

#### Conservation status.

The distribution area, population size and possible threats to the habitat of *Hoya ruthiae* are not known, as it is only known from the type specimen and unlocalised cultivated material. It is therefore considered Data Deficient (DD) (IUCN 2014).

#### Notes.

The most striking feature of *Hoya ruthiae* is its lack of coloured latex. Other species lacking coloured latex are the type species of the genus *Hoya carnosa* R.Br. and several morphologically similar taxa (Rodda and Simonsson Juhonewe 2012). All these have rotate corollas with thickly pubescent lobes within and rhomboid corona lobes with narrow inner and outer corona lobe processes. In Borneo only *Hoya monetteae* T. Green belongs to this group. *Hoya ruthiae*, as mentioned above, is morphologically very similar to *Hoya uncinata*, another non-laticiferous species known to occur in Java and Sumatra. Both species have deeply lobed rotate corollas, laterally compressed corona lobes and corpusculum of the pollinarium almost as large as the pollinium. *Hoya ruthiae* has larger flowers (1.8–2.2 cm in diameter when flattened vs. 1.5–1.7 cm) and the corolla lobes are narrowly lanceolate (vs. ovate in *Hoya uncinata*). The corona lobes of both species are ovate but in *Hoya ruthiae* the outer process is round while in *Hoya uncinata* it terminates in an incurved membranaceous apiculate appendage. Flowers of *Hoya uncinata* are illustrated in Fig. 2.

**Figure 1.**
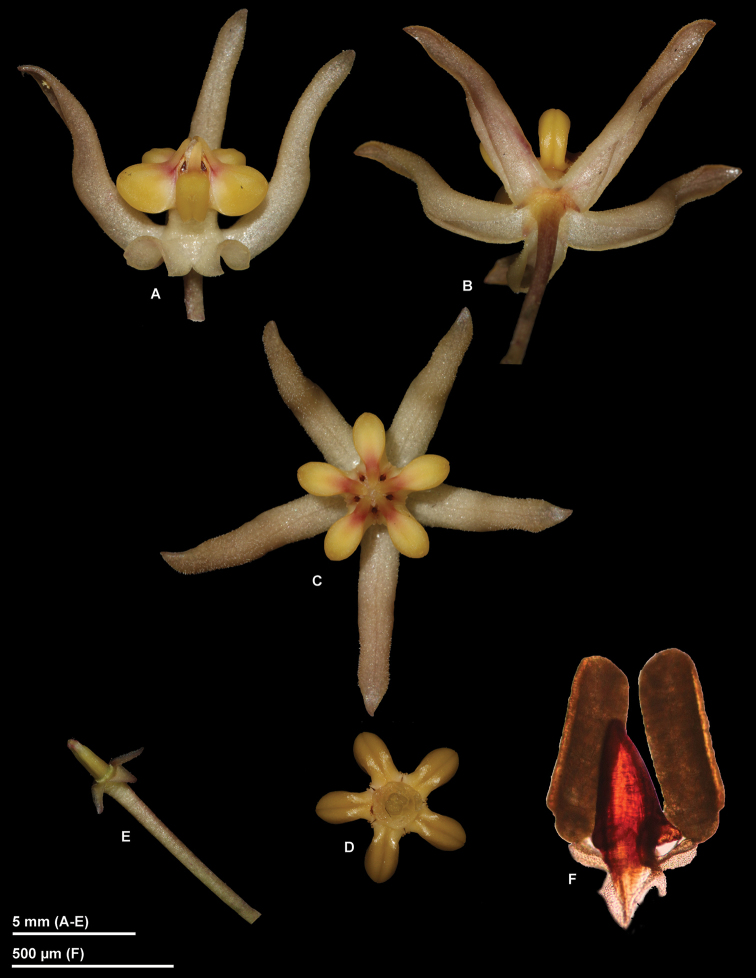
*Hoya ruthiae* photographed from *Rodda M. MR606* (SING) prior to pressing **A** Flower, lateral view with two corolla lobes removed **B** Corolla, underneath **C** Corolla and corona, top view **D** Corona, underneath **E** Pedicel, calyx and ovaries **F** Pollinarium with twin pollinia. (Photographs by M. Rodda)

**Figure 2.**
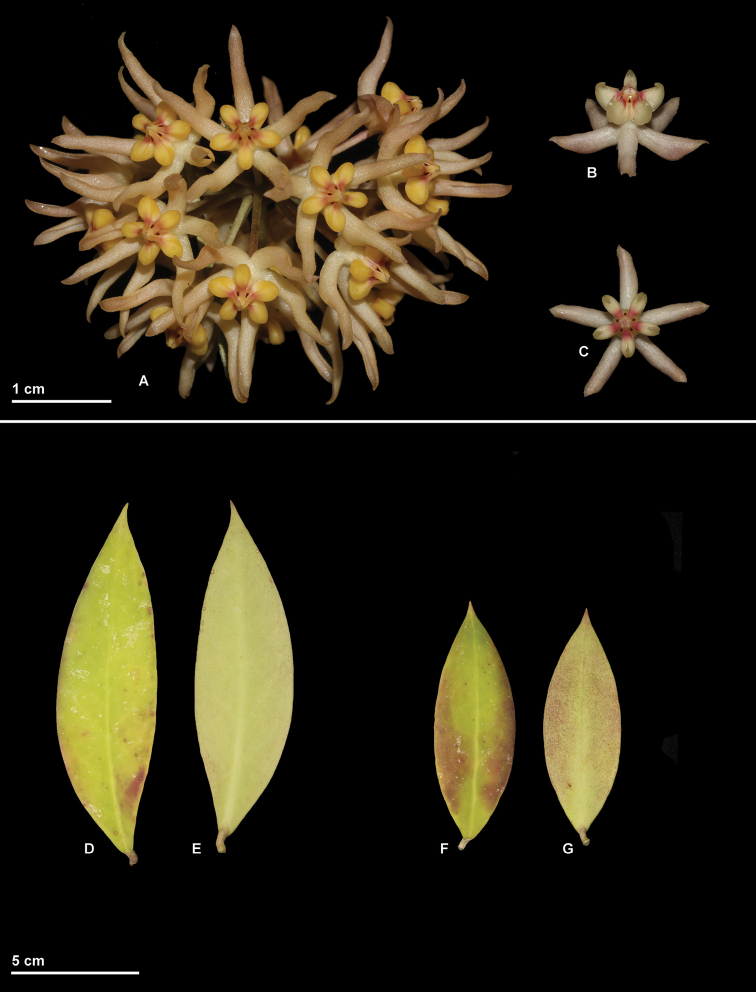
*Hoya ruthiae* photographed from *Rodda M. MR606* (SING) prior to pressing **A** Inflorescence **D, E, F, G** Two leaves (**D, F** adaxial surface **E, G** abaxial surface). *Hoya uncinata* photographed from *Rodda M MR607* (SING) prior to pressing **B** Flower, lateral view **C** Flower, top view. (Photographs by M. Rodda)

#### Additional specimens examined.

Unlocalised (nursery origin), Cultivated in Thailand, Ratchaburi Prov., Ratchaburi, 23 March 2014, *Rodda M MR606* (SING).

### Hoya bakoensis

Taxon classificationPlantaeGentianalesApocynaceae

Rodda sp. nov.

urn:lsid:ipni.org:names:77148381-1

Figs 3 , 4 , 5

#### Diagnostic characters.

Similar to *Hoya aeschynanthoides* Schltr. as both species have bilobed outer corona lobes, but differing in the size and colour of the flowers (3.5–4 mm in diameter, pink corolla, yellow and red corona lobes for *Hoya bakoensis*; ca. 5 mm in diameter and white for *Hoya aeschynanthoides*) and in the habit, that in *Hoya aeschynanthoides* is decumbent while *Hoya bakoensis* is a weak twining climber.

#### Type.

Malaysia, Borneo, Sarawak, Bako National Park, along Tajor Trail, 20 March 2015, *Rodda M MR1042b* (SING, holotype; SAR, KEP, isotypes)

#### Description.

Epiphytic climber with white exudate in all vegetative parts. *Stems* slender, weakly twining upward, internodes (1 mm–)2–7(–10) cm × 0.7–1.5(–2) mm, dull green, sparsely pubescent when young, scabrous; *adventitious root* sparsely produced along the stems and just under the nodes where they are paired. *Leaves* petiolate; petiole straight or recurved, 4–10 × 1–1.5 mm, dark green to maroon, sparsely pubescent; *lamina* ovate (lanceolate) (1.5–)2–4(–5) × 1–2.8 cm, base attenuate (acute), apex apiculate (cuspidate), dark green above, slightly pubescent on young leaves only, lighter green underneath with occasionally a slightly darker midrib and edge, glabrous, margin occasionally ciliate; penninerved, secondary veins obscure; *colleters* one at each lamina base, triangular to ovate 0.1–0.3 × 0.3–0.5 mm. *Inflorescence* pseudo-umbelliform, slightly convex, 10–15 flowered; *peduncle* 4–6 cm × 1–1.5 mm in diameter, dull green to brown, pubescent; rachis indeterminate (–7) × ca. 2 mm in diameter. *Pedicel* 3–7 × 0.7–0.9 mm in diameter, pale green with pink spots, papillose. *Calyx* lobes triangular, 0.7–1 × 0.5–0.8 mm, apex acute or round, light green with pink edge, sparsely ciliate; *basal colleter* one in each calyx lobe sinus, ovate, 150–200 × 80–100 µm. *Corolla* revolute, 3.5–4 mm in diameter, ca. 6 mm when flattened; *corolla lobes* basally fused, tube 1.2–1.5 mm long, lobes triangular, 2–3 × 2.4–2.7 mm, pink, pubescent inside, outside glabrous. *Corona* staminal, 2.8–3 mm in diameter, 0.9–1.1 mm high; *corona lobes* oblong, 1.2–1.4 × 0.7–0.8 mm, convex above, underneath sulcate with inrolled margins, outer process apex bifid, light yellow, inner process elongate, red, with a yellow round tip. *Anthers* broadly triangular, 400–450 × 550–650 µm, with apical triangular membranaceous appendage. *Pollinia* clavate, 200–250 × 90–110 µm, narrowing towards the base, apex obliquely truncate, with evident pellucid margin; *corpusculum* oblong, constricted in the middle, 80–100 × 50–60 µm; *caudicle* broad, spathulate, hyaline, 110–130 × 30–45 µm at the widest. *Style-head* 5 angled in cross section, with 5 lobes alternating with the stamens, style-head apex round, 200–300 µm long, ca. 0.5 mm broad at the base; *ovary* ovate with a narrow tip, 0.8–1 mm, each carpel ca. 0.3 mm wide at the base, light green. *Fruit* (unripe) a single linear follicle, 10 cm × 2 mm (widest), *seed* (unripe) narrowly lanceolate, 3–4 mm long, winged, long comose.

#### Etymology.

The species is named after the collection locality of the holotype, Bako National Park (Sarawak, Malaysia).

#### Distribution and ecology.

*Hoya bakoensis* is only known from Bako National Park, where it is common along Tajor Trail in moist, shady kerangas heath forest near a stream. The species is strictly epiphytic and it was found mostly germinating from the opening of small ant nests located inside hollow tree trunks (Fig. 4). The plants observed were forming small clumps of weakly climbing stems near the germinating point or more rarely were climbing towards the tree canopy potentially in response to low light.

**Figure 3.**
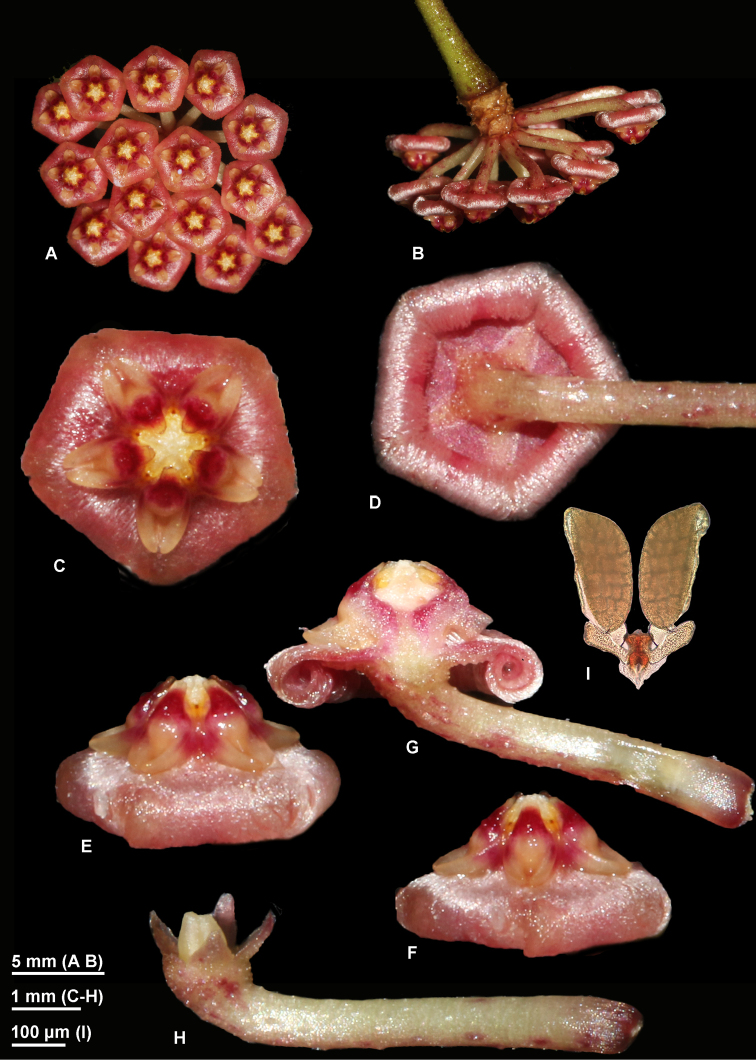
*Hoya bakoensis* photographed in the field from the type plant *Rodda M MR1042b* (SING) prior to pressing. **A** inflorescence, frontal view **B** Inflorescence, side view **C** A single flower, front view **D** Revolute margins of the corolla lobes, calyx and pedicel **E, F** Corolla, side view **G** Flower, longitudinal section **H** Pedicel, calyx and ovary **I** Pollinarium with twin pollinia. (Photographs by M. Rodda)

#### Conservation status.

*Hoya bakoensis* is locally common and well protected inside Bako National Park. Its conservation status is therefore Least Concern (LC) (IUCN 2014).

#### Notes.

*Hoya bakoensis* is one of the 11 Bornean *Hoya* species in Hoya section Acanthostemma (Bl.) Kloppenb. *Acanthostemma* species are characterised by revolute corolla lobes, bilobed outer corona lobes and pollinaria with broad, spathulate caudicles. The other Bornean species in *Acanthostemma* are *Hoya aeschynanthoides*, *Hoya acicularis*, T. Green, *Hoya beccarii* Rodda & Simonsson, *Hoya kloppenburgii* T. Green, *Hoya minutiflora* Rodda and Simonsson, *Hoya pubera* Bl., *Hoya revoluta* Wight ex Hook.f., *Hoya rundumensis* (T. Green) Rodda and Simonsson, *Hoya sigillatis* T. Green and *Hoya waymaniae* Kloppenb.

Most of these species can be easily separated from *Hoya bakoensis* because their flowers are larger (>5 mm in diameter) or their inflorescence is markedly concave (in *Hoya beccarii* and *Hoya revoluta*). As mentioned in the diagnosis *Hoya aeschynanthoides* has white, only slightly larger flowers than *Hoya bakoensis* (ca. 5 vs. 3.5–4 mm in diameter). Additionally the bilobed outer lobes of *Hoya aeschynanthoides* appear to be less pronounced than those of *Hoya bakoensis*, but this observation is based solely on the examination of the drawing attached to the type of *Hoya aeschynanthoides* as only small buds are present on the specimen while no other specimens have been found at present. The flowers of *Hoya pubera* are also comparable in size with those of *Hoya bakoensis*, but they are yellow-orange, the corolla lobes are only apically revolute and the corona is more markedly raised in the centre. Lastly, *Hoya minutiflora* has the smallest flowers among all Bornean *Acanthostemma* (2.6–2.8 mm in diameter).

**Figure 4.**
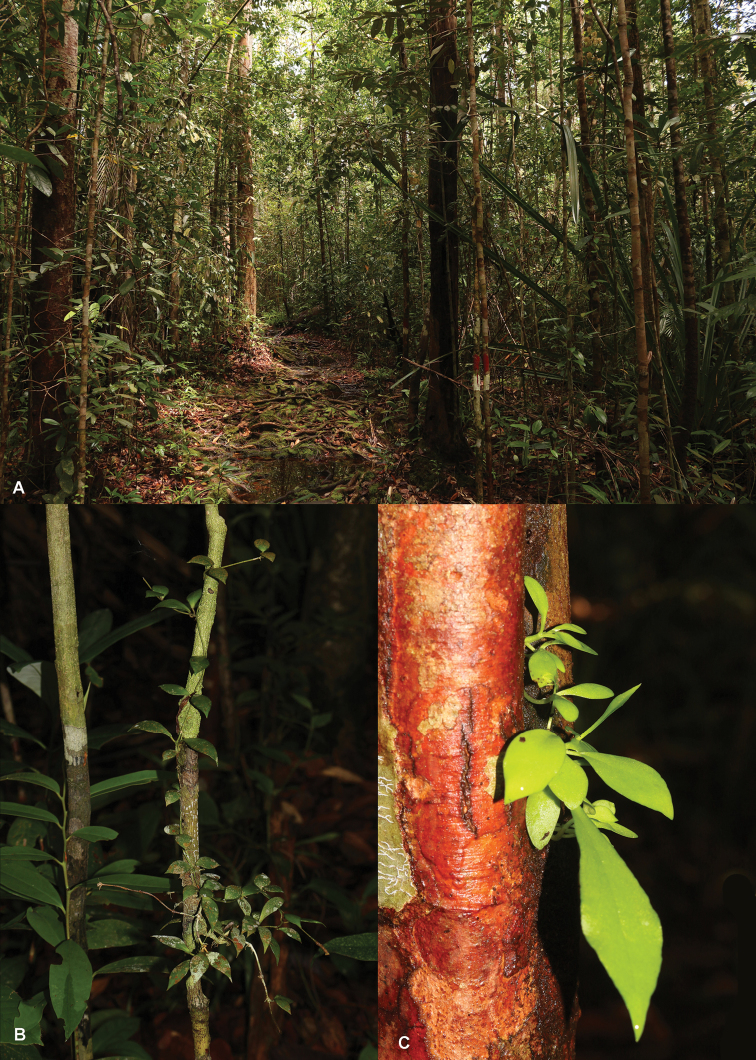
*Hoya bakoensis in situ* in Bako National Park (Sarawak, Malaysia) **A** Habitat, kerangas heath forest **B** Mature plant rooted inside the trunk of the host plant where an ant nest is located **C** Seedlings germinating from the opening of an ant nest in a hollow trunk.

**Figure 5.**
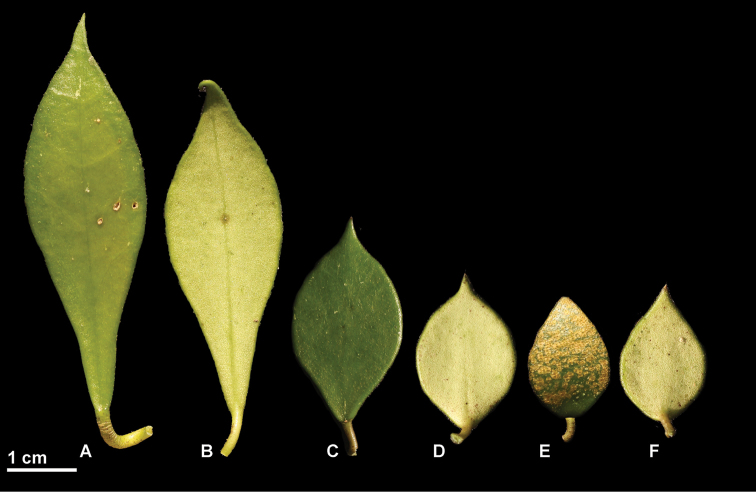
*Hoya bakoensis* leaves photographed in the field from *Rodda M MR1042b* (**A, B, E, F**) and *Rodda M MR1042a* (**C, D**) (SING) prior to pressing. **A, C, E** Adaxial side **B, D, F** Abaxial side.

#### Additional specimens examined.

Malaysia, Borneo, Sarawak, Bako National Park, along Tajor Trail, 20 March 2015, *Rodda M MR1042a* (KEP, SAR, SING)

## Supplementary Material

XML Treatment for Hoya ruthiae

XML Treatment for Hoya bakoensis
